# Continuous Epidural Analgesia Versus Continuous Peripheral Nerve Block in Unilateral Lower Extremity Pediatric Orthopedic Surgery: A Matched Case Comparison Study

**DOI:** 10.7759/cureus.40412

**Published:** 2023-06-14

**Authors:** Neeraj Vij, Neil R Singhal, Daniel Trif, Aaron Llanes, Ali Fanharawi, Matt Pankratz, Sanjana Khanna, Mohan Belthur

**Affiliations:** 1 Orthopedic Surgery, University of Arizona College of Medicine - Phoenix, Phoenix, USA; 2 Anesthesiology, Phoenix Children's Hospital, Phoenix, USA; 3 Anesthesiology, University of Texas at San Antonio, San Antonio, USA; 4 Pediatric Orthopedics, Phoenix Children's Hospital, Phoenix, USA; 5 Pediatric Orthopedics, University of Arizona College of Medicine - Phoenix, Phoenix, USA

**Keywords:** postoperative pain control, pediatric regional anesthesia, continuous epidural analgesia, pediatric lower extremity surgery, continuous peripheral nerve block

## Abstract

Introduction

Continuous epidural analgesia (CEA) provides effective postoperative pain relief but includes a substantial side effect profile. Continuous peripheral nerve blocks (CPNBs) have fewer side effects and may quicken ambulation. The purpose of this study was to compare the morphine milligram equivalents (MMEs), need for analgesic rescue, visual analog scale (VAS) pain scores, time to ambulation, postoperative blood pressures, length of stay (LOS), and adverse event rates.

Methods

This was a matched case comparison study of pediatric patients (ages 8-17) undergoing unilateral lower limb surgery (41 CEA and 36 CPNB). Patients with a history of chronic pain, previous lower extremity surgery, and developmental delay were excluded. The Chi-square test and Student’s t-test were used, and p-values < 0.05 were considered significant.

Results

There were no statistically significant differences in demographics or the American Society of Anesthesiologists (ASA) grade. There were no significant differences in postoperative MMEs, the need for analgesic rescue, or VAS scores on any postoperative day. The CEA group had a longer time to ambulation (2.56 ± 0.93 days versus 1.89 ± 0.69 days, p = 0.004). The CEA group demonstrated a higher number of days of systolic hypotension (0.61 ± 0.97 mmHg versus 0.06 ± 0.23 mmHg, p = 0.0009) and diastolic hypotension (1.90 ± 1.24 mmHg versus 1.00 ± 0.93 mmHg, p = 0.0006). There were no significant differences in the length of stay between the CEA and CPNB groups (5.08 versus 4.24, p = 0.28). There was no statistically significant difference between the rates of pruritus, light-headedness, and altered mental status. The CEA group demonstrated higher rates of nausea (51.2% versus 13.9%, p = 0.001), constipation (36.6% versus 8.3%, p = 0.004), urinary retention (9.8% versus 0%, p = 0.006), and average number of minor adverse events per patient (1.02 versus 0.25, p = 0.002).

Conclusions

CPNBs and CEAs demonstrate equivalent postoperative opioid use after unilateral lower extremity surgery in the pediatric population. In our population, a low complication rate and a decreased time to ambulation were seen in the CPNB group. There may be certain select scenarios priorly managed with a CEA that can be appropriately managed with a CPNB. A prospective multicenter study incorporating patient satisfaction data could further facilitate the incorporation of CPNB in pediatric pain management protocols after orthopedic surgery.

## Introduction

Practices regarding pediatric anesthesia have changed in recent years [1]. Several advances have affected the current practice surrounding regional anesthesia [2]. This includes advances in ultrasound guidance [3,4], nerve mapping [5], and anesthetic choice. These have facilitated the incorporation of single-shot and continuous peripheral nerve blocks (CPNBs) for pain relief after pediatric surgery. The increase in the use of CPNBs within pediatric anesthesia has correlated with a decrease in neuraxial anesthesia [2].

Alternative options for postoperative pain control after pediatric orthopedic surgery include a single-shot regional nerve block, single-shot caudal analgesia, or continuous epidural analgesia (CEA). However, epidural analgesia comes with a significant side effect profile including nausea, vomiting, and urinary retention. There is also a high complication rate after epidural analgesia, including cardiac morbidity, pulmonary embolism, and acute renal failure [6]. Side effects are noticeably higher in the pediatric population [7]. A recent study reported an incidence of 7.6 in 1,000 for a major complication (life-threatening injury or significant neurologic deficit), which is more than 10 times higher than the major complication rate in adults [8].

CPNBs are effective in providing adequate pain-free days and decreasing reliance on opioids in the postoperative period [9-12]. There has been much excitement regarding the increased utilization of CPNBs after pediatric orthopedic surgery, although clinical efficacy studies are lacking [13,14].

It is well known that perineural analgesia is a safe modality for pain control in the pediatric population [3,9,15,16]. An analysis of 100,000 blocks from the Pediatric Regional Anesthesia Network (PRAN) demonstrated a very low complication rate with no sequelae lasting greater than three months [16]. CPNBs have also demonstrated good patient satisfaction scores in the pediatric population [17]. However, there are limited reports regarding the clinical efficacy of the CPNB in terms of pain scores and postoperative opioid requirements [12].

As the literature around CPNB grows, surgical teams have become more comfortable using CPNB as the mainstay of postoperative pain control [13,14,18]. There are many clinical scenarios in which CPNB would not provide adequate postoperative analgesia and thus may warrant a form of neuraxial anesthesia. However, as the evidence behind CPNB increases, there may be clinical scenarios priorly controlled with a CEA that may be amenable to CPNB as a promising alternative. The purpose of this study is to compare CPNB to CEA after lower extremity pediatric orthopedic surgery. The primary outcome of this study was the postoperative morphine milligram equivalents (MMEs). The secondary outcomes were the need for analgesic rescue, VAS pain scores, time to ambulation, postoperative blood pressures, length of stay (LOS), and adverse event rates. We hypothesized that there would be no difference in MMEs, need for analgesic rescue, VAS pain scores, or LOS between the groups and that the CPNB group would have a shorter time to ambulation, decreased rates of postoperative hypotension, and decreased adverse event rates.

## Materials and methods

General

This was a retrospective, matched case comparison study at a large tertiary referral center that was approved by our Institutional Review Board (IRB number 16-053). The patient charts for all patients receiving lower extremity orthopedic surgery in consultation with the pain service were searched and divided into the following categories: single-shot epidural, continuous epidural; single-shot nerve block; and continuous nerve block. Pediatric patients (ages 0-18) who received unilateral lower extremity orthopedic surgery were included. Patients from the single-shot groups, patients undergoing bilateral lower extremity surgery, and patients with a history of chronic pain, previous lower extremity surgery, and developmental delay were excluded. The CPNBs used in our study included lumbar plexus (4), femoral (8), popliteal (17), and femoral + popliteal (7). The patients included in the continuous peripheral nerve block (CPNB) group were age- and gender-matched to the patients in the continuous epidural analgesia (CEA) group.

Data collection and management

Data was collected using Microsoft Excel version 16.66.61 (Microsoft Corp., Redmond, WA, USA) and stored in an encrypted folder on our institution’s secure network between October 1, 2014, and December 31, 2021. The demographic variables obtained included gender, age, height, and weight. Surgical information obtained included the date, details of the surgery, and ASA grade. Postoperative pain information collected included the choice of continuous analgesic modality, continuous medication given, length of use, daily morphine usage, other opioid medications given, any non-opioid medications given, and length of stay (LOS). The need for analgesic rescue (defined as a requirement for any as-needed analgesic medication) was also recorded [19]. All minor adverse events [13] (infection, pruritus, nausea/vomiting, urinary retention, constipation, overdose, or compartment syndrome) and major adverse events [13] (permanent neurologic injury or mortality) were gathered. Systolic and diastolic blood pressures immediately postoperatively and on postoperative day 1 were recorded. The number of days of systolic/diastolic hypotension was also recorded. MMEs were calculated using the daily pro re nata (PRN) and patient-controlled analgesia (PCA) [20]. The visual analog scale (VAS), which has demonstrated excellent validity in the age ranges 8-18 [13,21], was used to collect pain scores within the 0-12 hour, 12-24 hour, 24-36 hour, and 36-48 hour postoperative periods. The postoperative days of rehabilitation initiation and ambulation were also recorded.

Data abstraction and statistical analysis

Continuous variables were reported as means and standard deviations (SDs) and categorical variables as frequencies and proportions. A preliminary power analysis was performed, and it was determined that to detect a mean difference of MMEs that is equal to its standard deviation, 35 patients would need to be recruited to each group to achieve a statistical power of greater than 90%. The Chi-square test was used to compare continuous variables. Student’s t-test was used to compare categorical variables. SAS version 9.4 (SAS Institute Inc., Cary, NC, USA) was used for all statistical analyses. P-values were two-tailed, and statistical significance was set at p < 0.05.

## Results

General

There were a total of 35 males and 42 females in our study with an average age of 11.32 (± 2.80) years. There were no statistically significant differences in gender, age, body mass index (BMI), or ASA grades between the two groups (Table 1).

**Table 1:** Our population demographics and the ASA grade for our study groups ASA: American Society of Anesthesiologists, CEA: continuous epidural analgesia, CPNB: continuous peripheral nerve block

Variable	CEA group (n = 41)	CPNB group (n = 36)	P-value
Males	18, 43.9%	17, 47.2%	0.77
Age	11.02 ± 2.80	11.67 ± 2.81	0.32
Body mass index	23.68 ± 8.47	24.35 ± 13.19	0.8
ASA grade 1	22 (53.7)	14 (38.9)	0.41
ASA grade 2	13 (31.7)	16 (44.4)	
ASA grade 3	6 (14.6)	6 (16.7)	

**Figure 1:**
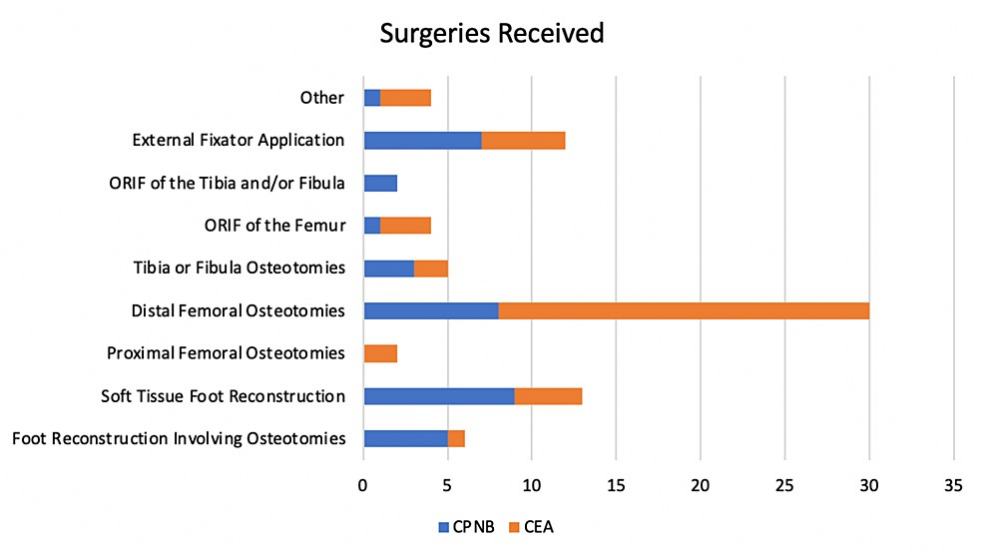
Surgeries received by the CPNB and CEA groups The “Other” group consisted of two patients who received excision and curettage of a femoral lesion and one patient who received a hamstring lengthening procedure (CEA group) and one patient who received a subtalar arthrodesis (CPNB group). One CPNB and two CEA patients from the femoral osteotomy groups concurrently received a distal femoral hemiepiphysiodesis for the treatment of miserable malalignment. CEA: continuous epidural analgesia, CPNB: continuous peripheral nerve block

The surgeries performed in the CPNB and CEA groups can be seen in Figure 1.

Opioid use and analgesic rescue

There were no significant differences in MMEs required on any postoperative day (measured up to postoperative day 3) between the two groups (Table 2).

**Table 2:** Total postoperative morphine milligram equivalent requirements (mg/kg) CEA: continuous epidural analgesia, CPNB: continuous peripheral nerve block

Postoperative day	CEA group (n = 41)	CPNB group (n = 36)	P-value
0	0.116 ± 0.206	0.065 ± 0.021	0.19
1	0.179 ± 0.188	0.129 ± 0.113	0.15
2	0.115 ± 0.017	0.112 ± 0.019	0.92
3	0.076 ± 0.086	0.066 ± 0.101	0.65

There were no significant differences in the need for analgesic rescue at any point in the hospital stay between the CEA and CPNB groups (80.4% versus 86.1%, p = 0.88).

VAS pain scores

There were no statistically significant differences in pain scores between the CEA and CPNB groups in any postoperative period (Table 3).

**Table 3:** VAS pain scores after surgery VAS: visual analog scale, CEA: continuous epidural analgesia, CPNB: continuous peripheral nerve block

Postoperative period (hours)	CEA group (n = 41)	CPNB group (n = 36)	P-value
0-12	1.16 ± 1.69	1.14 ± 1.95	0.98
12-24	2.11 ± 1.78	1.46 ± 1.33	0.08
24-36	1.85 ± 1.75	1.61 ± 1.45	0.52
36-48	1.69 ± 1.54	1.28 ± 1.41	0.23

Rehabilitation and ambulation

The CEA group had a longer time to rehabilitation initiation (1.89 ± 0.86 days versus 1.15 ± 0.78 days, p = 0.0003) and a longer time to ambulation (2.56 ± 0.93 days versus 1.89 ± 0.69 days, p = 0.004) than the CPNB group (Table 4).

**Table 4:** Comparison of rehabilitation initiation and ambulation between our groups Two patients in both groups denied therapy. Fourteen (34.2%) patients and eight (22.2%) patients from the CEA and CPNB groups did not ambulate during their hospital stay. CEA: continuous epidural analgesia, CPNB: continuous peripheral nerve block, SD: standard deviation

	Postoperative day	CEA group (n = 41)	CPNB group (n = 36)	P-value
Rehabilitation initiation	0	2 (5%)	4 (11.1%)	0.0002
	1	10 (25%)	24 (66.7%)	
	2	16 (40%)	4 (11.1%)	
	3	10 (25%)	1 (2.8%)	
	4	0	1 (2.8%)	
	Overall (average, SD)	1.89 ± 0.86	1.15 ± 0.78	0.0003
Day of ambulation	0	1 (2.4%)	0	0.006
	1	1 (2.4%)	7 (19.4%)	
	2	10 (24.4%)	18 (50%)	
	3	13 (31.7%)	2 (5.6%)	
	4	1 (2.4%)	1 (2.8%)	
	5	1 (2.4%)	0	
	Overall (average, SD)	2.56 ± 0.93	1.89 ± 0.69	0.004

A total of eight patients in the CPNB group did not ambulate during their hospital stay. This included four distal femoral osteotomy patients, two foot reconstruction with osteotomy patients, and two soft tissue foot reconstruction patients. A total of 14 patients in the CEA group did not ambulate during their hospital stay. This included one proximal femoral osteotomy patient, 12 distal femoral osteotomy patients, and one patient who received an excision and curettage of a femoral lesion.

Blood pressure

The CEA group demonstrated a higher number of days of systolic hypotension (0.61 ± 0.97 mmHg versus 0.06 ± 0.23 mmHg, p = 0.0009) and diastolic hypotension (1.90 ± 1.24 mmHg versus 1.00 ± 0.93 mmHg, p = 0.0006) than the CPNB group (Table 5).

**Table 5:** Comparison of blood pressure data between our groups Systolic hypotension was defined as <120 mmHg, and diastolic hypotension was defined as <90 mmHg [16]. CEA: continuous epidural analgesia, CPNB: continuous peripheral nerve block

	Postoperative day	CEA group (n = 41)	CPNB group (n = 36)	P-value
Systolic hypotension	0	27 (65.9%)	34 (94.4%)	0.01
	1	6 (14.6%)	2 (5.6%)	
	2	5 (12.2%)	0	
	3	3 (7.3%)	0	
	Overall (days)	0.61 ± 0.97	0.06 ± 0.23	0.0009
Diastolic hypotension	0	5 (12.2%)	13 (36.1%)	0.04
	1	12 (29.3%)	12 (33.3%)	
	2	11 (26.8%)	9 (25%)	
	3	9 (22%)	2 (2.6%)	
	4	3 (7.3%)	0	
	5	1 (2.4%)	0	
	Overall (days)	1.90 ± 1.24	1.00 ± 0.93	0.0006

Length of stay

There were no significant differences in the length of stay between the CEA group and the CPNB group (5.08 versus 4.24, p = 0.28) (Table 6).

**Table 6:** Comparison of length of stay between the two groups CEA: continuous epidural analgesia, CPNB: continuous peripheral nerve block, SD: standard deviation

Length of stay (days)	CEA group (n = 41)	CPNB group (n = 36)	P-value	Overall (N = 77)
<3	2 (4.9)	3 (8.3)	0.2	5 (6.5)
3-4	24 (58.5)	21 (58.3)		45 (58.4)
5-9	15 (36.6)	9 (25)		24 (31.2)
>10	0	3 (8.3)		3 (3.9)
Overall (average, SD)	5.08 ± 4.4	4.24 ± 1.30	0.28	4.64 ± 3.21

Adverse events

There were no major adverse events in either group. There was no statistically significant difference between the rates of pruritus, light-headedness, and altered mental status between the CEA and CPNB groups (Table 7).

**Table 7:** Comparison of adverse events between the two groups CEA: continuous epidural analgesia, CPNB: continuous peripheral nerve block

Minor adverse event	CEA group (n = 41)	CPNB group (n = 36)	P-value	Overall (N = 77)
Pruritus	2 (4.9%)	1 (2.8%)	0.63	3 (3.9%)
Nausea	20 (51.2%)	5 (13.9%)	0.001	25 (32.5%)
Constipation	15 (36.6%)	3 (8.3%)	0.004	18 (23.4%)
Urinary retention	4 (9.8%)	0	0.006	4 (5.2%)
Light-headedness	1 (2.4%)	0	0.35	1 (1.3%)
Altered mental status	1 (2.4%)	0	0.35	1 (1.3%)
Average number of minor adverse events per patient	1.02	0.25	0.0002	0.66

The CEA group demonstrated higher rates of nausea (51.2% versus 13.9%, p = 0.001), constipation (36.6% versus 8.3%, p = 0.004), urinary retention (9.8% versus 0%, p = 0.006), and average number of minor adverse events per patient (1.02 versus 0.25, p = 0.002).

## Discussion

This matched case comparison study identified 41 and 36 patients in whom CEA and CPNB were the primary forms of postoperative analgesia, respectively. Both groups encompassed a similar spectrum of lower extremity orthopedic surgeries. We demonstrated no difference in postoperative MMEs, need for analgesic rescue, VAS scores, or LOS. The CPNB group demonstrated a shorter time to ambulation, decreased postoperative hypotension, and a decreased minor adverse event rate.

It is important to consider postoperative opioid use when considering the choice of regional anesthesia in children undergoing orthopedic surgery [22,23]. There has been much conversation regarding the reduction of opioid use after pediatric orthopedic surgery [24,25]. Opioids are often overprescribed [14] and underutilized [25] after pediatric orthopedic surgery. Further, the harsh side effects of opioids are seen in higher numbers in children [3]. However, postoperative pain management protocols in orthopedics generally do not take into account the variety of multimodal options available to patients, and perineural techniques remain underused [26]. The results of our study favor more frequent utilization of CPNBs in children undergoing unilateral lower extremity orthopedic surgery. There are certain scenarios in which a CPNB may be insufficient, and ultimately, the decision about using a CEA or alternative regional anesthesia should be made based on the involvement of both extremities, intensiveness of the procedure, surgical site, and family/patient preferences.

There is good data to suggest a low adverse event rate with the use of continuous peripheral nerve blocks. The published literature demonstrates a minor adverse event rate of 17% and a nausea/vomiting rate of 14.7% [12,13]. This correlated well with our minor adverse event rate of 25% and nausea/vomiting rate of 13.9%. This is in contrast to the nausea/vomiting rate of 51.2% seen in our CEA population, which also coincides with the literature [8]. The safety and practice patterns analysis from the PRAN demonstrated a minor adverse event rate of 12.1% [27], although notably, their large-scale study did include other side effects including persistent neurologic problems, infection, and local anesthetic systemic toxicity (LAST) that were also not seen in our review of 77 patients. CPNBs are not without their limitations, including inadequate pain relief in the proximal extremities, limitations to patients undergoing unilateral surgery, and reliance on a pain team. Nevertheless, in the appropriate clinical scenario, the low adverse event rate seen in our study does demonstrate some favorability.

A recently published article highlights the aforementioned points. Laron et al. [22] demonstrated that in patients with cerebral palsy undergoing hip reconstructive surgery, the fascia iliaca block provides decreased postoperative pain scores and decreased opioid usage than continuous lumbar epidural analgesia. These encouraging results are in line with the findings of our study. An interesting consideration is whether more of the femoral osteotomy patients in our study could be managed more appropriately with a fascia iliaca block. In our study, the two proximal femoral osteotomy patients received a CEA. Nonetheless, the results of Laron et al. [22] are encouraging, and further studies should aim to determine the efficacy of peripheral analgesic options in hip surgery in children.

In the published literature, local analgesia proves to be as successful or even better than epidural analgesia in select scenarios. Novais et al. [23] demonstrated equivalent postoperative pain scores and opioid consumption in patients who received periarticular local infiltrative analgesia as compared to those who received epidural analgesia in the context of surgical hip dislocation for the treatment of femoroacetabular impingement. While the results of this study are specific to a select patient population and thus the clinical scenarios warranting epidural analgesia remain plentiful, this study further highlights the role of local and regional analgesic options in pediatric patients undergoing orthopedic surgery.

There have been several recent advances within regional anesthesia that further make CPNB an attractive option in the right clinical scenario. This includes nerve mapping and advances in ultrasound techniques that allow for better visualization in children [4]. Perineural catheters can be placed with increasing ease in children [28], which further makes CPNBs an attractive option. At our institution, nerve blocks are placed by board-certified pediatric anesthesiologists under direct sonographic visualization without nerve mapping. In our study, this yielded a low complication rate similar to that seen in the published literature [13,27].

It is well known that early mobilization leads to improved functional outcomes after orthopedic surgery [29,30]. Early mobilization leads to decreased complications in children undergoing pediatric orthopedic surgery [30]. In our study, the majority (66.7%) of the CPNB group began rehabilitation on the first postoperative day and were ambulatory (50%) by the second postoperative day. It is important to keep in mind that the patient’s perceived mental strength and the physical therapist’s perception of the patient’s health may have influenced the earlier time to ambulation seen in the CPNB group. Our ability to draw conclusions on this result is very limited due to the heterogeneity of operations and their effect on ambulatory status. The association between CPNB and early mobilization should be explored in future studies.

Limitations

This was a non-randomized, retrospective study and is thus subject to inherent limitations. Although our study groups were age- and gender-matched and did demonstrate similarity in ASA grades, there is an element of heterogeneity in terms of procedures performed. Our study took place at a single tertiary referral pediatric hospital with a highly trained pain service, and thus, our results may not be generalizable. Certain pain practitioners may have a bias toward one nerve block over another. Lastly, our sample size of 77 patients was chosen to power our study with regard to MMEs; thus, there is a risk of type II statistical error concerning the secondary outcomes.

## Conclusions

CPNBs and CEAs demonstrate equivalent postoperative opioid use after unilateral lower extremity surgery in the pediatric population. In our population, a low complication rate and a decreased time to ambulation were seen in the CPNB group. There may be certain select scenarios priorly managed with a CEA that can be appropriately managed with a CPNB. A prospective multicenter study incorporating patient satisfaction data could further facilitate the incorporation of CPNB in pediatric pain management protocols after orthopedic surgery.
